# Cyclosporine A, in Contrast to Rapamycin, Affects the Ability of Dendritic Cells to Induce Immune Tolerance Mechanisms

**DOI:** 10.1007/s00005-021-00632-7

**Published:** 2021-10-10

**Authors:** Maja Machcińska, Monika Kotur, Aleksandra Jankowska, Marta Maruszewska-Cheruiyot, Artur Łaski, Zuzanna Kotkowska, Katarzyna Bocian, Grażyna Korczak-Kowalska

**Affiliations:** 1 Department of Immunology, Institute of Functional Biology and Ecology, Faculty of Biology, University of Warsaw, Warsaw, Poland; 2 Laboratory of Parasitology, General Karol Kaczkowski Military Institute of Hygiene and Epidemiology, Warsaw, Poland; 3 Present Address: Present address: Laboratory of Parasitology, General Karol Kaczkowski Military Institute of Hygiene and Epidemiology, Warsaw, Poland

**Keywords:** Dendritic cells, Immune tolerance, T cells, Immunosuppressive agents, Rapamycin, Cyclosporine A

## Abstract

Following organ transplantation, it is essential that immune tolerance is induced in the graft recipient to reduce the risk of rejection and avoid complications associated with the long-term use of immunosuppressive drugs. Immature dendritic cells (DCs) are considered to promote transplant tolerance and may minimize the risk of graft rejection. The aim of the study was to evaluate the effects of immunosuppressive agents: rapamycin (Rapa) and cyclosporine A (CsA) on generation of human tolerogenic DCs (tolDCs) and also to evaluate the ability of these cells to induce mechanisms of immune tolerance. tolDCs were generated in the environment of Rapa or CsA. Next, we evaluated the effects of these agents on surface phenotypes (CD11c, MHC II, CD40, CD80, CD83, CD86, CCR7, TLR2, TLR4), cytokine production (IL-4, IL-6, IL-10, IL-12p70, TGF-β), phagocytic capacity and resistant to lipopolysaccharide activation of these DCs. Moreover, we assessed ability of such tolDCs to induce T cell activation and apoptosis, Treg differentiation and production of Th1- and Th2-characteristic cytokine profile. Data obtained in this study demonstrate that rapamycin is effective at generating maturation-resistant tolDCs, however, does not change the ability of these cells to induce mechanisms of immune tolerance. In contrast, CsA affects the ability of these cells to induce mechanisms of immune tolerance, but is not efficient at generating maturation-resistant tolDCs.

**Supplementary Information:**

The online version contains supplementary material available at 10.1007/s00005-021-00632-7.

## Introduction

Following organ transplantation, it is essential that immune tolerance is induced in the graft recipient to reduce the risk of rejection and avoid complications associated with the long-term use of immunosuppressive drugs. It is hence crucial to develop methods to promote donor-specific tolerance in the recipient. Although existing therapies are focused on effector cells, a great deal of attention is being paid to dendritic cells (DCs), which may promote transplant tolerance (Marín et al. 2016; Svajger et al. 2014).

Owing to their capacity to regulate both the innate and adaptive immune responses, DCs are considered to play crucial roles in directing the alloimmune response towards transplant tolerance or rejection. It is known that immature DCs, also known as tolerogenic DCs (tolDCs), may minimize the risk of graft rejection and promote transplant tolerance (Li and Shi 2015; Sanjana et al. 2017). Transplant tolerance itself comprises a number of distinct mechanisms, such as the induction of T cell anergy and apoptosis, the induction of T regulatory cell (Tregs) proliferation and the selective activation of Th2 cells (Horton et al. 2017; Ochando et al. 2020). These properties enable tolDCs to be used as a base for therapeutic strategies to promote organ transplant tolerance (Moreau et al. 2017). An approach combining tolDCs with various immunosuppressive agents can ensure long-term allograft survival or transplant tolerance (Que et al. 2020; Švajger and Rožman 2020; Thomson et al. 2018, 2019).

Two of the most commonly and effectively used immunosuppressive drugs used after organ transplantation are rapamycin (Rapa) and cyclosporine A (CsA) (Enderby and Keller 2015; Moini et al. 2015). For many years, their main targets were thought to be T cells; however, it has recently been found that immunosuppressive agents could also inhibit DC maturation and allostimulatory capacity (Cangemi et al. 2019; Macedo et al. 2012). Not only has this provided a new insight into the immunopharmacology of these substances, but it also offers novel strategies for the manipulation of DCs ex vivo prior to organ transplantation to promote tolerance induction. There is currently a global effort to create cellular therapies using tolDCs and immunosuppressive drugs to improve long-term outcomes in organ transplantation (Marín et al. 2016; Moreau et al. 2017; Thomson et al. 2019); despite this, little is known about the effects of these drugs on human DCs and past studies lack a unified approach to tolDC generation protocols, and there remains a great need for more comprehensive studies. In addition, previous studies have not examined such a broad range of effects exerted by DCs generated in the environment of immunosuppressive drugs (Rapa and CsA) on the activation and function of T cells.

Considering that the ultimate goal of such research is to transfer tolDC-based therapy from the laboratory to the clinic, it is extremely important not only to generate DCs with stable tolerogenicity, which are resistant to an exogenous maturation stimulus, but also, and perhaps above all, produce tolDCs which are able to induce mechanisms of immune tolerance. Therefore, the present study evaluates the tolerogenic surface phenotype, anti-inflammatory cytokine production, phagocytic capacity and resistance to maturation of monocyte-generated DCs to determine their tolerogenic properties; it also examines their effects on T cell activation and survival, Treg induction and selective activation of Th2 cells.

## Materials and Methods

### Generation of Monocyte-Derived DCs

Human peripheral blood mononuclear cells (PBMC) were isolated from the fresh buffy coats of 30 healthy donors from Regional Centre for Blood Donation and Treatment in Warsaw, Poland by density gradient centrifugation using Ficoll-Paque (Gradisol L; Aqua Medica, Lodz, Poland). The donors met the requirements of the Regional Blood stations and agreed to provide blood for research purposes.

The monocytes were isolated from PBMC using an EasySep Human CD14 Positive Selection Kit (StemCell Technologies, Seattle, WA, USA) according to the manufacturer’s instructions. The purity, determined by flow cytometry (FACSVerse, Becton Dickinson) using CD14-PE monoclonal antibody (clone 61D3; eBioscience, USA) post isolation, was greater than 95%.

For DC differentiation, monocytes were cultured at 1 × 10^6^/ml in AIM-V (Gibco/Invitrogen, Breda, Netherlands) culture medium supplemented with 200 U/ml rhGM-CSF (R&D Systems, USA) and 500 U/ml rhIL-4 (R&D Systems) on 24-well plates at 37 °C, 5% CO_2_ for 5 days (DC, Control DC). To generate immature DCs with tolerogenic properties, 200 ng/ml CsA (CsA-DC; Novartis Pharma, Germany) or 20 ng/ml Rapa (Rapa-DC; Wyeth-Lederle Pharma, UK) was added from the beginning of cultures. Immature DCs were harvested at day 5 and cell viability was determined using trypan blue (> 96% for each sample).

### Activation of Monocyte-Derived DCs

To induce maturation, 1–1.5 × 10^6^/ml immature DCs, Rapa-DC and CsA-DC were stimulated with 1 μg/ml lipopolysaccharide (LPS) from *E. coli* O26:B6 (Sigma–Aldrich, USA). Cells are referred as DC + LPS, Rapa-DC + LPS and CsA-DC + LPS, respectively. Additionally, Control DCs were also activated with LPS, together with 20 ng/ml Rapa (DC + LPS + Rapa) or 200 ng/ml CsA (DC + LPS + CsA). Cells were incubated on 24-well plates for 24 h (37 °C, 5% CO^2^) and viability was > 97% for each sample.

### Phenotypic Characterisation by Flow Cytometry

The immature and mature DCs were subjected to phenotypic characterization by flow cytometry analysis. The following fluorochrome-conjugated monoclonal antibodies were used according to the manufacturers’ protocols: CD11c-APC (clone 3.9; eBioscience, USA), CD14-PE (clone 61D3; eBioscience, USA), CD1c-FITC (clone L161; eBioscience, USA), CD40-PE (clone 5C3; BD Pharmingen, USA), CD80-PE (clone 2D10.4; eBioscience, USA), CD86-PE-Cy7 (clone IT2.2; eBioscience, USA), CD83-FITC (clone HB15E; BD Pharmingen, USA), MHC II-eFluor450 (clone L243; eBioscience, USA), CCR7-APC-eFluor780 (clone 3D12; eBioscience), TLR2-FITC (clone TL2.1; eBioscience, USA), TLR4-PE (clone HTA125; eBioscience, USA), CD36-FITC (clone CD38; eBioscience, USA), DEC205-PE (clone MG38; BD Pharmingen, USA).

**Fig. 1:**
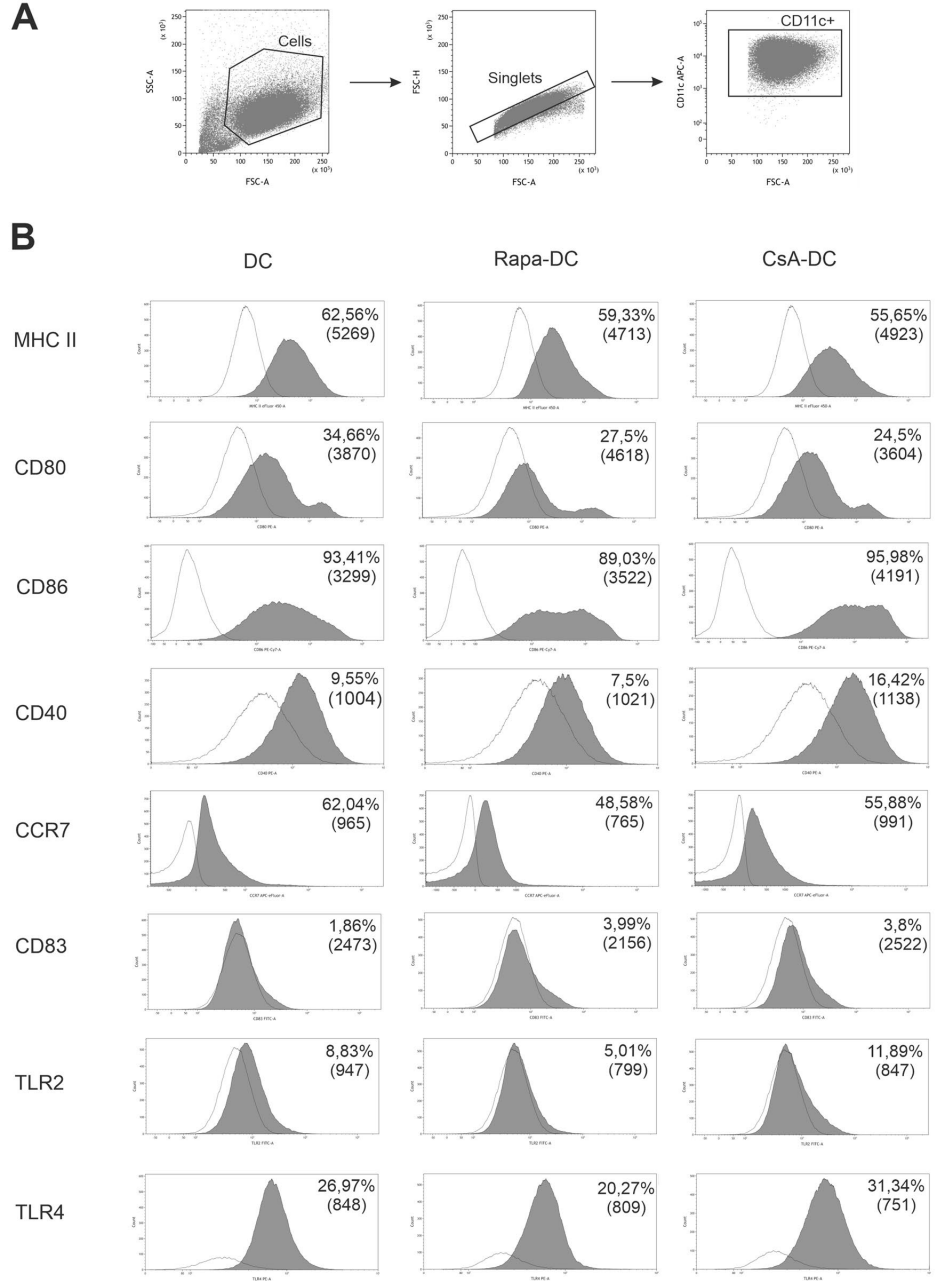
Effects of Rapa and CsA on the expression of DCs surface markers. Immature DCs were differentiated in the environment of immunosuppressive agents: Rapa (Rapa-DC) or CsA (CsA-DC) and without drugs (DC). Gating strategy for flow cytometric analysis of DCs: a time gate was initially applied to exclude any electronic noise and artifact (not shown here). Next, based on size and granularity, DCs were gated in a forward scatter area (FSC-A) versus side scatter area (SSC-A) plot. Then, doublet cells were excluded using FSC-A/FSC-height (FSC-H) parameters. Within the singlet cell population CD11c^+^ DCs were gated (**A**), followed by expression of individual markers shown on representative histograms for DC, Rapa-DC and CsA-DC (**B**). The averages of the percentage of positively labelled CD11c^+^ DCs and mean fluorescence intensity in bracket 7–14 different donors are reported on each histogram. The white shade indicates FMO control. Representative dot plots are presented

Briefly, the cells (1 × 10^6^) were resuspended in 100 μl Cell Wash (BD Bioscience, USA) and incubated with appropriate monoclonal antibodies for 30 min in the dark at 4 °C. Following this, the cells were washed twice with Cell Wash and acquired on FACSVerse calibrated daily using BD FACSuite CS&T Research Beads Kit (BD Bioscience, USA). The results were analyzed using FACSuite software (Becton Dickinson, USA) and Kaluza Analysis Software (Beckman Coulter, USA). Single-stained cells or BD CompBead Plus (BD Bioscience, USA) were used for compensation in all assays. The positive staining and gating strategy were determined by comparison with an unstained control and fluorescence minus one (FMO) control, if applicable. The DC population was identified based on morphological parameters on a forward vs side scatter (FSC-A/SSC-A) plot. Cell aggregates were removed from the analysis using FSC-A versus FSC-H parameters. Dendritic cells were defined as CD11c^+^ cells (Fig. 1). In all experiments, at least 100,000 events were analyzed for each sample. The results were shown as the percentage of positively labelled cells and the mean fluorescence intensity (MFI) was calculated by FACSVerse.

### Measurements of Cytokine Production

Supernatants from immature and mature DC cultures were collected and the presence of interleukin (IL)-6, IL-10, IL-12p70, and transforming growth factor (TGF)-β was analysed. Supernatants collected from MLR at day 5 of culture were analysed for the presence of Th2 cytokines: IL-4, IL-5, IL-10, IL-13 and Th1 cytokines: IL-2, interferon (IFN)-γ. The concentration of cytokines was quantified using ELISA Ready-Set-Go! assay (eBioscience, USA) according to the manufacturer’s instructions. The absorbance was measured at 450 nm with a microplate autoreader μQuant (BioTek) and analyzed using Gen5 Data Analysis Software (BioTek). Because IL-4 was added to the culture medium to induce the differentiation of monocytes into DCs, its expression in immature and mature DCs was determined by intracellular staining. The cells were incubated on 24-well plates for 4 h (37 °C, 5% CO2) in the presence of 3 μg/ml brefeldin A (eBioscience, USA), and were then washed twice with PBS and stained with Fixable Viability Dye eFluor506 (eBioscience, USA) and CD11c-APC according to the manufacturer’s instructions. The cells were then washed with Cell Wash, fixed and permeabilised using Intracellular Fixation and Permeabilization Buffer Set (eBioscience, USA) and incubated for 20 min at room temperature with IL-4-PE (clone 8D4-8; eBioscience, USA). The cells were then washed and analysed by flow cytometry.

### FITC-Dextran Uptake Assay

Immature DC, Rapa-DC and CsA-DC (1 × 10^6^) were incubated with FITC-dextran (molecular weight: 40,000; Sigma–Aldrich, USA) at the final concentration of 1 mg/ml for 30 min at 37 °C (control plate at 4 °C); the mixture was then washed extensively with Cell Wash and subjected to CD11c-APC staining for flow cytometry analysis.

### Allogenic Mixed Lymphocyte Reaction

Allogenic mixed lymphocyte reaction (MLR) cultures were created by culturing 1 × 10^6^ PBMCs, freshly isolated by density gradient, with 1 × 10^5^ DC, Rapa-DC or CsA-DC (10:1 ratio) in 200 μl AIM-V culture medium in 96-well, round-bottom plates for 5 days (37 °C, 5% CO_2_). These mixed cultures are referred as DC:T, Rapa-DC:T and CsA-DC:T, respectively. The DCs were extensively washed after incubation with Rapa or CsA to avoid carry over of immunosuppressive agents to the MLR culture.

### Analysis of T Cell Activation

The T cells were labelled with 5 μM carboxyfluorescein succinimidyl ester (CFSE; Sigma–Aldrich, USA) according to the manufacturer’s instructions, and then cocultured with DC, Rapa-DC or CsA-DC. After 5 days of coculture, the cells were harvested and labelled with the following fluorochrome-conjugated monoclonal antibodies: CD3-APC (clone: OKT3, eBioscience, USA), CD69-FITC (clone: FN50, eBioscience, USA) and CD25-PE (clone: BC96, BD Pharmingen, USA) to identify T cells or CD11c-APC and PD-L1-FITC (clone: MIH1, BD Pharmingen, USA) to identify DCs. All procedures were performed according to the manufacturers’ protocols. The cells were prepared for flow cytometry analysis and analysed as described above.

### Analysis of T Cell apoptosis

The cells were harvested on day 5 of MLR, and apoptosis was determined by staining cells with a combination of Annexin-V FITC with propidium iodide using the Annexin-V FITC Apoptosis Detection Kit (BD Pharmingen, USA), according to the manufacturers’ protocols. The cells were co-stained with CD3-APC. Additionally, cells were labelled with CD4-FITC (clone: RPA-T4, eBioscience, USA), CD8-APC (clone: RPA-T8, eBioscience, USA), CD95-PE (clone: DX2, BD Pharmingen, USA) or CD95L-PE (clone: NOK-1, BioLegend, USA) to identify T cells and CD11c-APC, CD95L-PE, Lineage Cocktail 1 (lin 1; CD3, CD14, CD16, CD19, CD20, CD56)-FITC (BD Pharmingen, USA) to identify DCs. The results were acquired and analyzed as described above.

### Analysis of Treg Induction

Treg were identified after 5 days of coculture with DC, Rapa-DC or CsA-DC by staining cells with the following fluorochrome-conjugated monoclonal antibodies according to the manufacturers’ protocols: CD3-PerCP (clone: SK7, BD Pharmingen, USA), CD4-FITC, CD8-APC, CD25-APC or PE (clone: 2A3, BD Pharmingen, USA), CD28-FITC (clone: CD28.2, BD Pharmingen, USA), CD127-eFluor450 (clone: eBioRDR5, eBioscience). Intracellular analysis of Foxp3-PE (clone: 236A/E7, eBioscience, USA) was performed after fixation and permeabilization, using the FoxP3/Transcription Factor Staining Buffer Set (eBioscience, USA). The results were acquired and analyzed as described above.

### Statistical Analysis

Results are given as means ± standard deviations (SD) for *n* samples per group. Statistical analysis was performed by the non-parametric Wilcoxon matched pair test using GraphPad Prism 8 software (GraphPad Software). A *p* value of < 0.05 was considered statistically significant.

## Results

### Effects of Rapa and CsA on DC Differentiation

To confirm whether Rapa or CsA affect the differentiation of DCs from monocytes, monocytes were cultured with granulocyte–macrophage colony-stimulating factor (GM-CSF) and IL-4 (DC; control DC) with Rapa (Rapa-DC) or CsA (CsA-DC). To identify immature monocyte-derived DCs, the CD1c^+^CD11c^+^CD14^–^ cells were analysed. The presence of CsA during the DC differentiation procedure resulted in a significantly lower percentage of CD1c^+^CD11c^+^CD14^–^ DCs compared to Rapa-DC and Control DC. In contrast, Rapa promoted the differentiation of monocyte-derived DCs (Fig. S1).

### Phenotype of Rapa-DC and CsA-DC

To determine the surface phenotype, the expression of markers associated with T cell activation (CD40, CD80, CD83, CD86 and MHC II), DC migration (CCR7) and activation (TLR2, TLR4) was determined. Compared to the Control DC, the Rapa-DC were not significantly phenotypically different but demonstrated a higher CD80 MFI. The CsA-DC showed a significantly lower percentage of CD80-positive cells, but higher percentages of CD86^+^ and CD40^+^ DCs and their MFI values. Both immunosuppressive agents significantly reduced the percentage of CD11c^+^CCR7^+^ DCs and significantly increased that of CD11c^+^CD83^+^ DCs. In contrast, only Rapa-DC showed a significantly lower expression of TLR2 and TLR4 (Fig. 1).

### Cytokine Production by Rapa-DC and CsA-DC

**Fig. 2:**
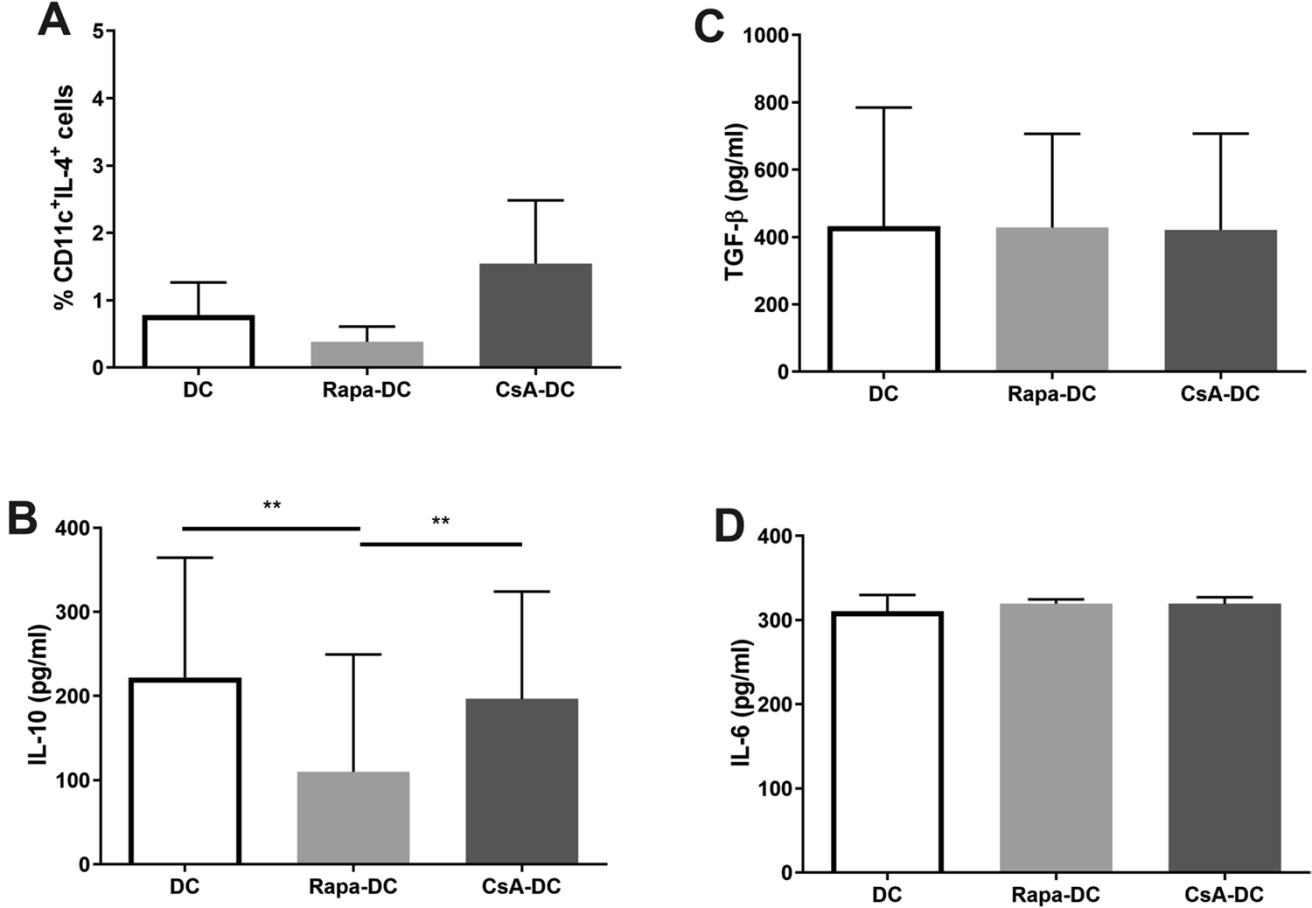
Effects of Rapa and CsA on the cytokine production by immature DCs. Immature DCs were differentiated in the environment of immunosuppressive agents: Rapa (Rapa-DC) or CsA (CsA-DC) and without drugs (DC). Cytokine expression was measured by intracellular staining (IL-4; **A**) or by ELISA of culture supernatants (IL-10, TGF-β and IL-6; **B**–**D**, respectively). Results are the averages ± SD of the percentage of positively labelled CD11c^+^ DCs (**A**; *n* = 5) or cytokine secretion (**B**–**D**; *n* = 19). **p ≤ 0.01; *p* values were calculated by Wilcoxon matched pair test

The production of IL-4, IL-10, TGF-β, IL-6 and IL-12p70 was compared between the DC types. Only IL-10 production was found to be lower in Rapa-DC than in DC and CsA-DC (Fig. 2). No significant changes in the production of other cytokines were associated with Rapa or CsA treatment during DC differentiation. In contrast, IL-12p70 was notably undetectable in all culture conditions (data not shown).

### The Phagocytic Capacity of Rapa-DC and CsA-DC

**Fig. 3:**
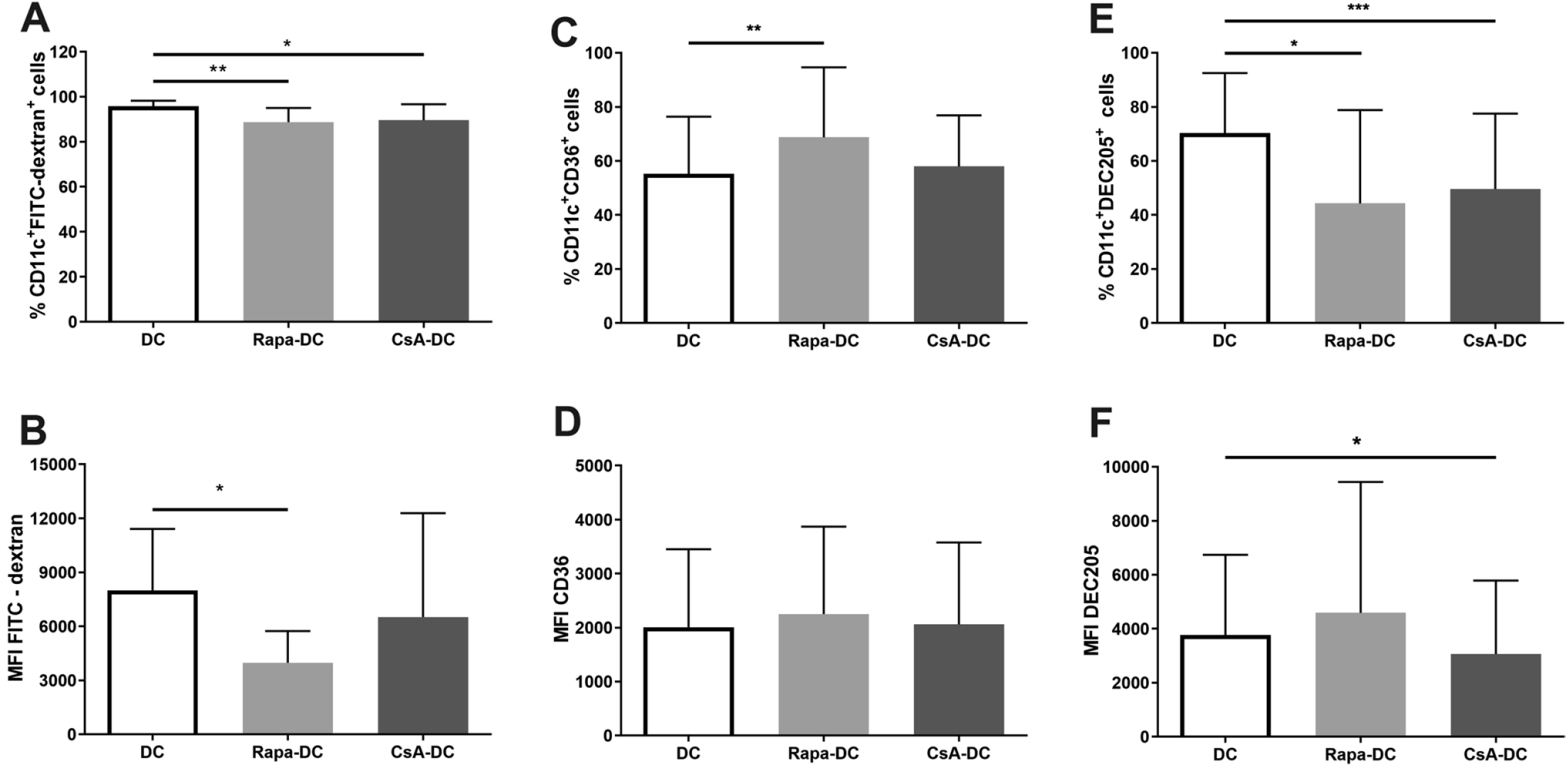
Effects of Rapa and CsA on phagocytic capacity of DCs. Immature DCs were differentiated in the environment of immunosuppressive agents: Rapa (Rapa-DC) or CsA (CsA-DC) and without drugs (DC). Cellular FITC-dextran uptake (**A**, **B**; *n* = 7) and the expression of phagocytosis receptors: CD36 (**C**, **D**; *n* = 10) and DEC205 (**F**, **G**; *n* = 11) were determined by flow cytometry. Results are the averages ± SD of the percentage of positively labelled CD11c^+^ DCs and mean fluorescence intensity (MFI). *p ≤ 0.05, **p ≤ 0.01, ***p ≤ 0.001; *p* values were calculated by Wilcoxon matched pair test

To determine the phagocytic capacity of Rapa-DC and CsA-DC, cellular FITC-dextran uptake was measured and the expression of phagocytosis receptors CD36 and DEC205 was determined by flow cytometry. The percentages of phagocytosing DCs, i.e. the percentage of CD11c^+^FITC-dextran^+^ cells, were significantly lower in the Rapa and CsA-treated DC culture compared to controls (Fig. 3A). Similar results were observed for FITC-dextran uptake by DCs (Fig. 3B).

A significantly higher percentage of CD11c^+^CD36^+^ was observed for Rapa-DC compared to Control DC. No significant differences in CD36 MFI value were found between the examined DCs. The presence of Rapa or CsA during DC differentiation significantly decreased the percentage of CD11c^+^DEC205^+^ DCs. However, significantly lower DEC205 MFI values were noted only in CsA-DC (Fig. 3C–F). Taken together these results suggest that Rapa-DC and CsA-DC demonstrate decreased phagocytic capacity compared to the Control DC.

### Effects of Rapa and CsA on DC Activation

To evaluate whether DCs differentiated in the environment with Rapa or CsA were resistant to an exogenous maturation stimulus, immature DCs were activated with LPS. The cell-surface phenotype and level of cytokine production were compared between immature and LPS-activated DCs to verify the process of DC maturation. After LPS activation, only the MFI values were significantly higher compared to immature DCs for all examined receptors. Additionally, significantly lower IL-10, TGF-β and IL-6 levels were observed in all LPS-activated DC culture supernatants compared with the immature cultures (data not shown).

### Phenotype of LPS-Activated Rapa-DC and CsA-DC

The maturation state of Rapa-DC and CsA-DC was determined by changes in the expression of surface markers. The percentage of LPS-activated DCs with CD11c^+^MHCII^+^CD80^+^CD86^+^CD83^+^CCR7^+^ phenotype was also determined; however, no significant differences were found between the Control DC group and the cultures treated with immunosuppressive agents (data not shown).

The Rapa-DC + LPS and CsA-DC + LPS groups demonstrated reduced, or comparable, expression of surface markers (both percentage positive cells and MFI values) to the control LPS-activated DCs. In particular, Rapa significantly decreased the percentage of DCs with MHC II and CD40 expression, as well as MHC II MFI values. CsA significantly decreased both the percentage of positive cells and MFI values of CD11c^+^MHC II^+^ and CD11c^+^CD80^+^ DCs. At the same time, Rapa-DC + LPS demonstrated a significantly higher CCR7 MFI value. In contrast, the presence of Rapa or CsA alone during LPS activation did not affect the expression of the examined markers in DCs (Table 1).

**Table 1:** Effects of Rapa and CsA on the expression of surface markers on LPS-activated DCs

Surface marker	Control		Immunosuppressive agents during differentiation				Immunosuppressive agents during activation			
	DC + LPS		Rapa-DC + LPS		CsA-DC + LPS		DC + LPS + Rapa		DC + LPS + CsA	
	%	MFI	%	MFI	%	MFI	%	MFI	%	MFI
MHC II	73.17 ± 13.13	9058 ± 4825	62.24 ± 19.33^a^	6985 ± 3594^a^	57.41 ± 18.86^a^	7632 ± 4345^a^	78.84 ± 13.01^b^	8747 ± 3738^b^	76.64 ± 13.74^c^	8508 ± 3423^c^
CD80	40.97 ± 20.70	7345 ± 4707	39.72 ± 29.90	8078 ± 4321	23.21 ± 14.53^a,b^	6630 ± 4067^a,b^	40.02 ± 19.33	6485 ± 3668	43.59 ± 19.92^c^	6178 ± 3105
CD86	95.44 ± 4.045	7333 ± 2713	97.16 ± 2.265	7286 ± 3136	94.34 ± 6.608	6677 ± 2953	96.94 ± 2.664	7190 ± 2620	97.22 ± 2.240	6568 ± 2075
CD40	8.180 ± 8.494	3319 ± 2037	5.118 ± 6.970^a^	3351 ± 2098	4.104 ± 3.101	3334 ± 2048	6.326 ± 5.899	3402 ± 1769	7.626 ± 7.449^d^	3320 ± 1726
CCR7	41.39 ± 13.60	1389 ± 1826	37.61 ± 23.70	2380 ± 3581^a^	41.79 ± 13.68	2513 ± 4701	39.41 ± 18.32	1199 ± 1538^b^	37.06 ± 13.72	1473 ± 2461^c^
CD83	1.889 ± 1.614	4358 ± 2672	4.848 ± 7.992	4863 ± 3394	3.556 ± 5.123^a^	4324 ± 2554	1.656 ± 1.103^a,b^	3994 ± 2255	2.149 ± 1.303^c^	3827 ± 2066
TLR2	5.475 ± 6.195	2173 ± 1636	3.968 ± 6.722	3475 ± 3128	5.328 ± 3.704	2931 ± 2237	2.982 ± 3.480	2732 ± 1814	5.043 ± 7.898	2604 ± 1684
TLR4	15.79 ± 24.20	2053 ± 1621	11.16 ± 19.02	2972 ± 2460	11.46 ± 13.82	2675 ± 2227	11.36 ± 20.00	2602 ± 1643	16.31 ± 28.18	2529 ± 1560

Immature DCs generated in the environment of: Rapa (Rapa-DC + LPS), CsA (CsA-DC + LPS) and without drugs (DC + LPS) were activated with LPS. Additionally, DCs, which differentiated without drugs, were activated with LPS simultaneously with Rapa (DC + LPS + Rapa) or CsA (DC + LPS + CsA). Surface markers expression was measured by flow cytometry. Results are the averages ± SD of the percentage of positively labelled CD11c^+^ DCs and mean fluorescence intensity (MFI) from 5 to 8 different donors

^a^Compared to DC + LPS (*p* < 0.05); ^b^compared to Rapa-DC + LPS (*p* < 0.05); ^c^compared to CsA-DC + LPS (*p* < 0.05); ^d^compared to DC + LPS + Rapa (*p* < 0.05); *p* values were calculated by Wilcoxon matched pair test

### Cytokine Production by LPS-Activated Rapa-DC and CsA-DC

**Fig. 4:**
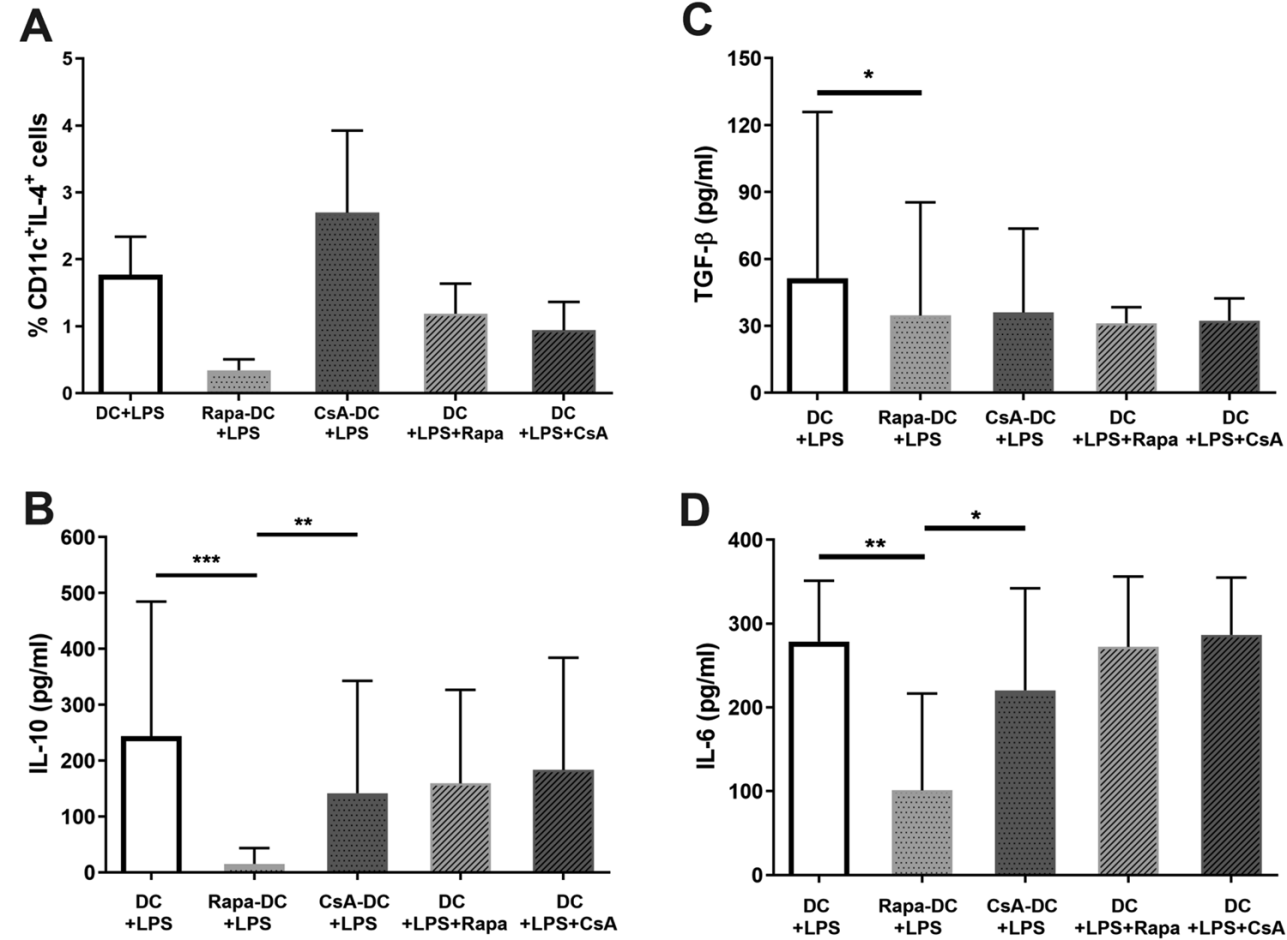
Effects of Rapa and CsA on the cytokine production by LPS-activated DCs. Immature DCs generated in the environment of: Rapa (Rapa-DC + LPS), CsA (CsA-DC + LPS) and without drugs (DC + LPS) were activated with LPS. Additionally, DCs, which differentiated without drugs, were activated with LPS simultaneously with Rapa (DC + LPS + Rapa) or CsA (DC + LPS + CsA). Cytokine expression was measured by intracellular staining (IL-4; **A**) or by ELISA of culture supernatants (IL-10, TGF-β and IL-6; **B**–**D**, respectively). Results are the averages ± SD of the percentage of positively labelled CD11c^+^ DCs (**A**) or cytokine secretion (**B**–**D**) from at least five different donors. *p ≤ 0.05, **p ≤ 0.01, ***p ≤ 0.001; *p* values were calculated by Wilcoxon matched pair test

The next stage examined the level of anti- and pro-inflammatory cytokine production by LPS-activated DCs. The results indicate no statistically significant differences in the percentage of CD11c^+^IL-4^+^ cells (LPS-activated DCs with intracellular IL-4 expression) between individual cultures; however, the percentage of these cells was almost eight times higher in CsA-DC + LPS than in Rapa-DC + LPS (Fig. 4A). Similar as for immature DCs, we observed a statistically significant lower levels of IL-10 and additionally IL-6 in supernatants of Rapa-DC + LPS compared to DC + LPS and CsA-DC + LPS and decrease TGF-β compared to control (Fig. 4B–D). IL-12p70 was notably undetectable in all culture conditions (data not shown). In addition, no significant differences in cytokine production were found when immunosuppressive agents were added simultaneously with LPS (i.e. DC + LPS + Rapa and DC + LPS + CsA).

### Effects of Rapa-DC and CsA-DC on T Cell Activation and Function

MLR cultures were performed to examine the induction of various mechanisms by Rapa-DC and CsA-DC. The levels of T cell activation markers, such as CD69 and CD25, were measured to determine the ability of Rapa-DC and CsA-DC to activate T cells. The results indicate that only the percentage of CD3^+^CD69^+^ cells in CsA-DC:T culture was significantly lower compared to controls. In addition, no significant differences in CD25 expression were observed (Fig. S2).

**Fig. 5:**
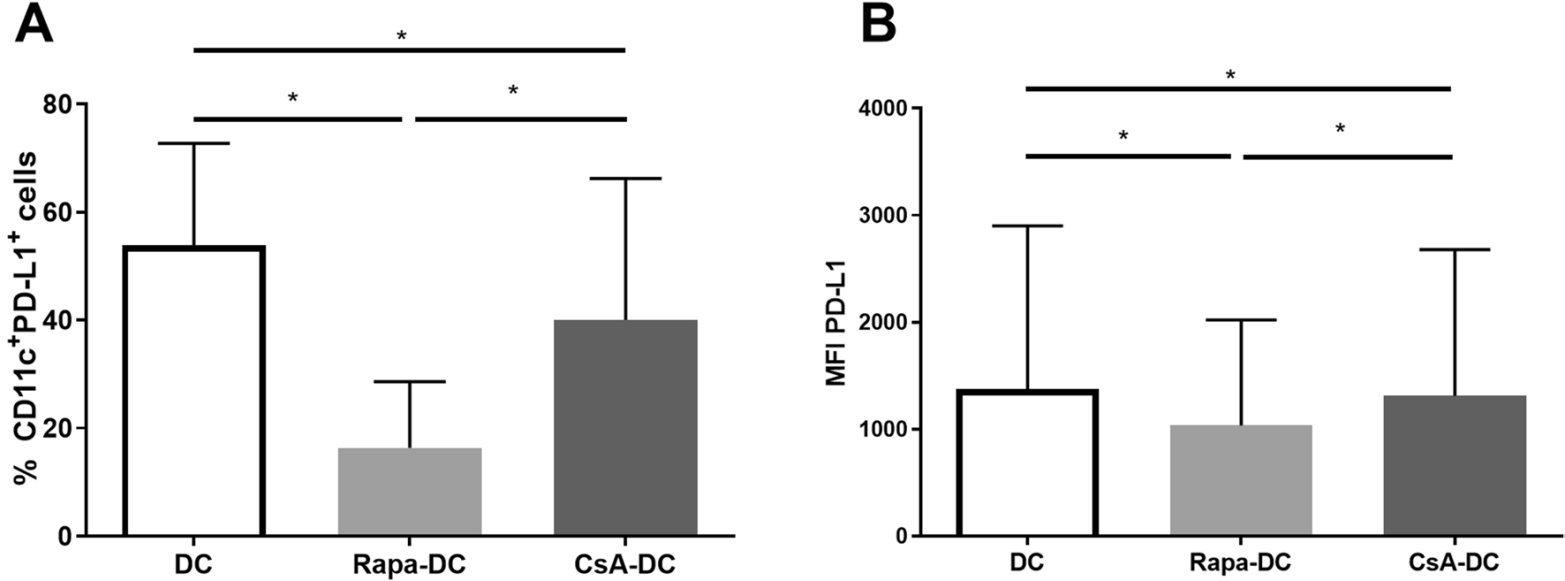
Effects of Rapa and CsA on PD-L1 expression on DCs. Immature DCs were differentiated in the environment of immunosuppressive agents: Rapa (Rapa-DC) or CsA (CsA-DC) and without drugs (DC). Expression of PD-L1 was determined by flow cytometry. Results are the averages ± SD of the percentage of CD11c^+^PD-L1^+^ DCs (**A**) and PD-L1 mean fluorescence intensity (MFI; **B**) from nine different donors. *p ≤ 0.05; *p* values were calculated by Wilcoxon matched pair test

To further investigate the effect of Rapa-DC and CsA-DC on T cell activation, T cell proliferation was assessed; the findings revealed no significant difference in the percentage of proliferation of T cells (data not shown). In addition, when Rapa or CsA was present during DC differentiation, significantly lower percentages of CD11c^+^PD-L1^+^ DCs and PD-L1 MFI values were noted for immature DCs (Fig. 5). PD-L1 inhibits T cell activation by interacting with its ligand PD-1 on T cells.

**Fig. 6:**
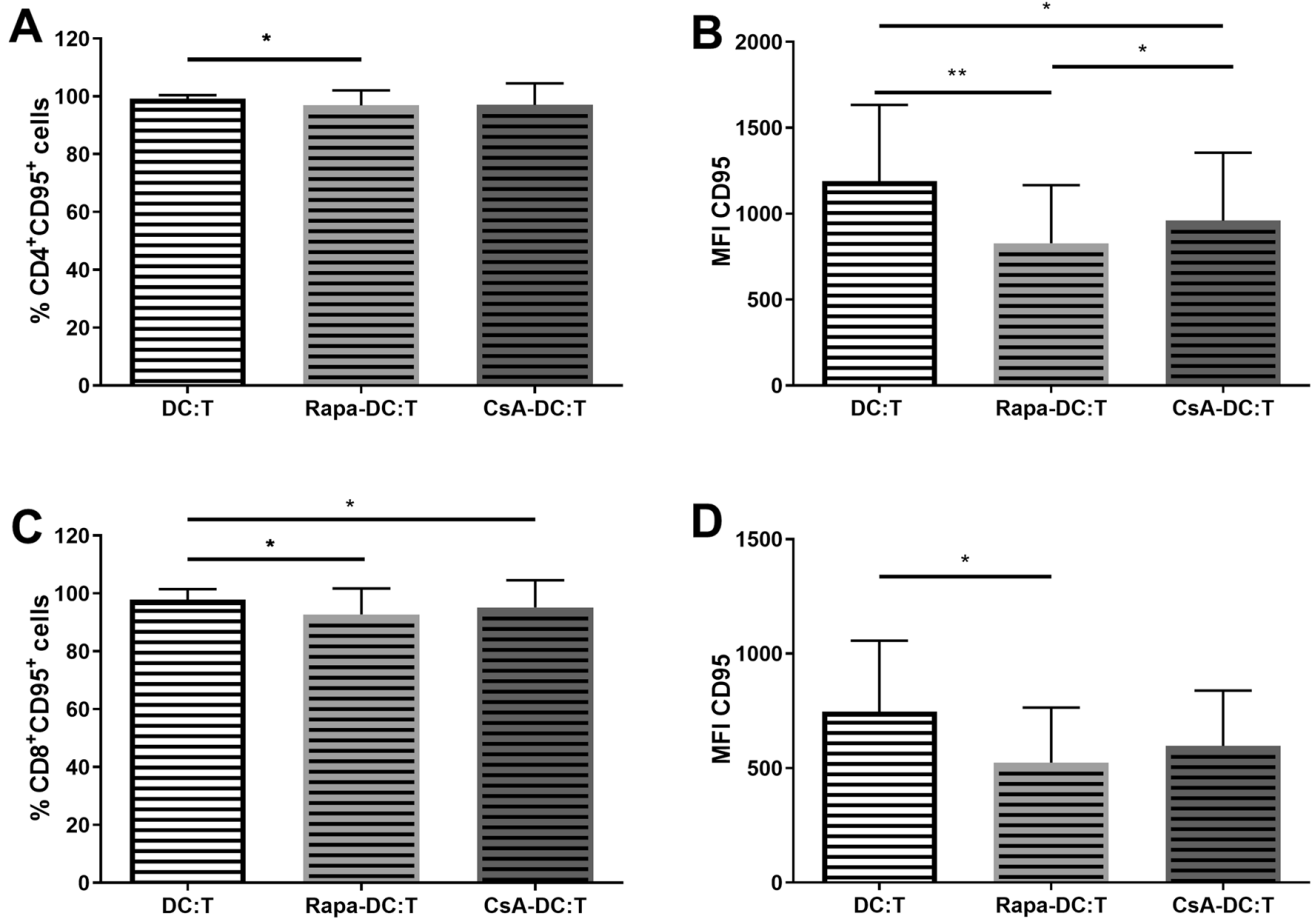
Effects of Rapa-DC and CsA-DC on the expression of CD95 on CD4^+^ and CD8^+^ T cells. Immature DCs, Rapa-DC and CsA-DC were cocultured with T cells. Expression of CD95 was determined by flow cytometry. Results are the averages ± SD of the percentage of positively labelled CD4^+^ (**A**) or CD8^+^ (**C**) T cells and mean fluorescence intensity (MFI) for CD4^+^ (**B**) or CD8^+^ (**D**) T cells, from ten different donors. *p ≤ 0.05, **p ≤ 0.01; *p* values were calculated by Wilcoxon matched pair test

To gain a deeper insight into the ability of Rapa-DC and CsA-DC to induce T cell apoptosis, the expression of two death-inducting receptors was analysed: CD95 (Fas) on T cells and CD95L (FasL) on DCs. It was found that for both the CD4^+^ and CD8^+^ T cell populations, the percentage of CD95-postive cells and MFI values were significantly lower in the Rapa-DC:T and CsA-DC:T cultures compared to the controls (Fig. 6). As CD95L expression may increase during T cell activation, this was also tested. Our results indicate a slight but statistically insignificant increase in the percentage of CD4^+^CD95L^+^ and CD8^+^CD95L^+^ in MLR cultures with Rapa-DC and CsA-DC compared to controls (data not shown). No significant differences were found in the percentage of lin^−^CD11c^+^CD95L^+^ DCs in MLR cultures. Only the MFI value of CD95L was significantly higher in the CsA-DC:T culture compared to Rapa-DC:T (Fig. S3). Finally, no statistically significant differences in the percentages of live, necrotic or apoptotic T cells were found between the Rapa-DC:T and CsA-DC:T cultures (data not shown).

**Fig. 7:**
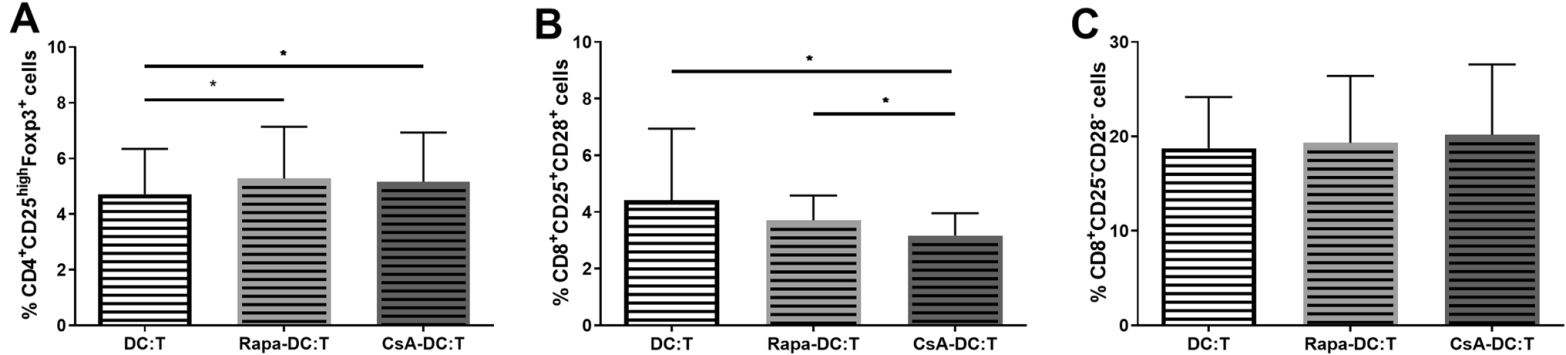
Effects of Rapa-DC and CsA-DC on T regulatory cells populations. Immature DCs, Rapa-DC and CsA-DC were cocultured with T cells. The percentage of CD4^+^CD25^high^Foxp3^+^ (**A**), CD8^+^CD25^+^CD28^+^ (**B**) and CD8^+^CD25^–^CD28^–^ (**C**) T cells were determined by flow cytometry. Results are the averages ± SD from ten different donors. *p ≤ 0.05; *p* values were calculated by Wilcoxon matched pair test

To determine whether the presence of Rapa-DC or CsA-DC in MLR cultures may increase the percentage of Treg cell populations, Tregs CD4^+^CD25^high^Foxp3^+^, CD8^+^CD25^+^CD28^+^ and CD8^+^CD25^–^CD28^–^ suppressor T cells were studied. After 5 days of MLR culture, a significant increase in the percentage of CD4^+^CD25^high^Foxp3^+^ Tregs was observed for both sets of T cells, i.e. those stimulated by Rapa-DC or by CsA-DC, compared to controls (Fig. 7A). In addition, the CD4^+^CD25^high^Foxp3^+^ Tregs demonstrated a significantly higher CD25 MFI value when cultured with Rapa-DC compared to controls and CsA-DC:T. Also, the Foxp3 MFI value was significantly higher in Tregs from Rapa-DC:T than CsA-DC:T (Fig. S4).

Then, as shown in Fig. 7, only the percentage of CD8^+^CD25^+^CD28^+^ T cells was significantly decreased in culture with CsA-DC, compared to controls and Rapa-DC. No differences were found in the CD25 and CD28 MFI values.

**Fig. 8:**
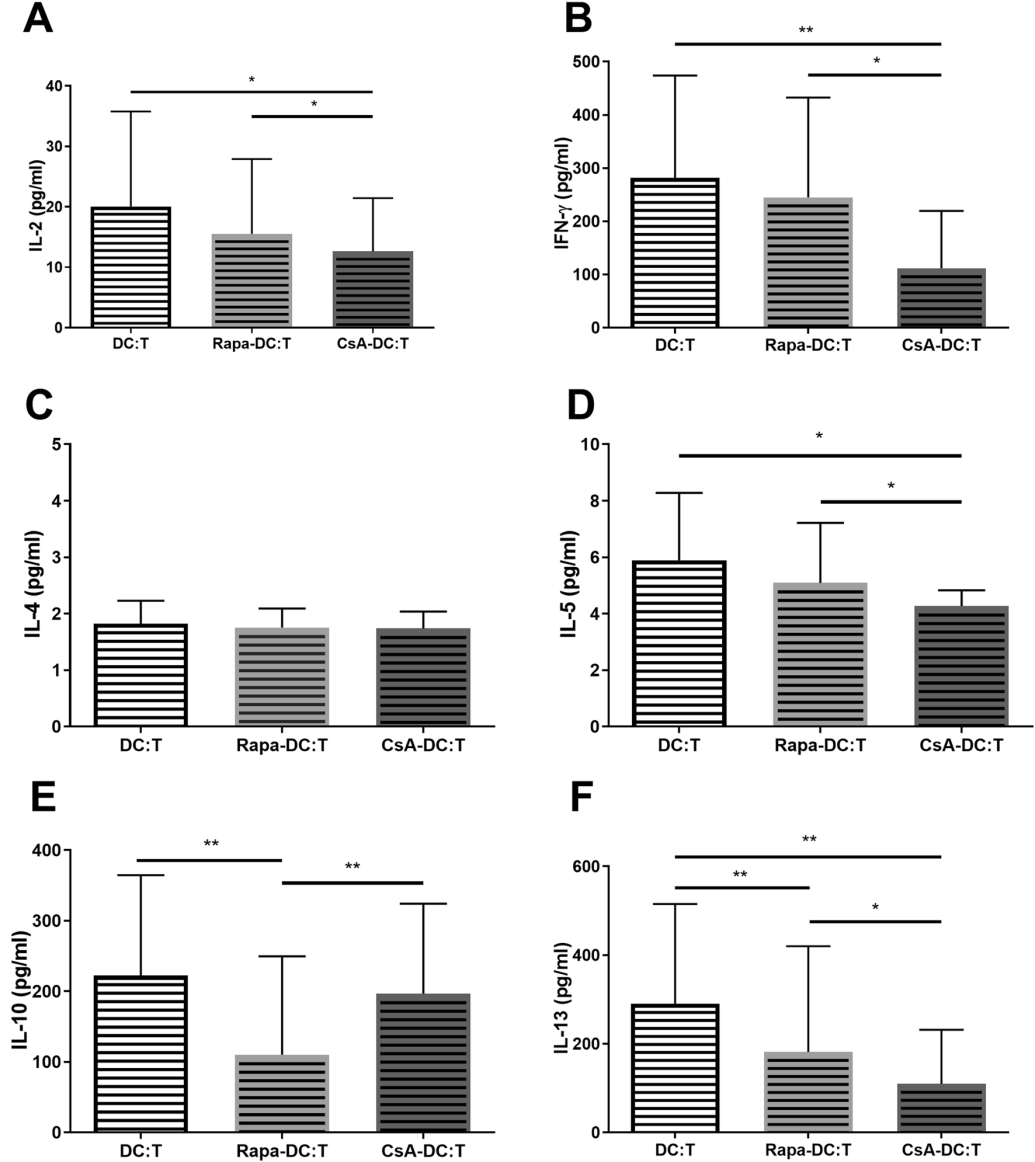
Effects of Rapa-DC and CsA-DC on the production of Th1- and Th2-characteristic cytokines. Immature DCs, Rapa-DC and CsA-DC were cocultured with T cells. Cytokine expression was measured by ELISA of culture supernatants for the presence of Th1 cytokines: IL-2 (**A**), IFN-γ (**B**) and Th2 cytokines: IL-4 (**C**), IL-5 (**D**), IL-10 (**E**), IL-13 (**F**). Results are the averages ± SD of cytokine secretion from 19 different donors. *p ≤ 0.05, **p ≤ 0.01; *p* values were calculated by Wilcoxon matched pair test

Finally, the production of a Th1- or Th2-characteristic cytokine profile was determined to assess Th2 cell activation. No significant differences were observed in IL-4 production by T cells under any culture conditions (Fig. 8C). However, significantly lower levels of other Th1 (IL-2, IFN-γ) and Th2 cytokines (IL-5, IL-10, IL-13) were detected in supernatants from T cells cocultured with Rapa- or CsA-DC (Fig. 8).

## Discussion

Possibly the greatest challenge faced by transplantation medicine in the twenty-first century is the induction of immune tolerance following organ transplantation. One important discovery in this regard is that tolDCs may not only induce transplant tolerance, but may also avoid the use of immunosuppressive agents and minimize immunosuppression-related side effects. Hence, attempts to develop tolDC-based therapies are required to improve long-term graft outcome.

Our findings indicate that the presence of Rapa during DC differentiation increased the percentage of CD1c^+^CD11c^+^CD14^–^ DCs, whereas CsA decreased it. These suggest that the environment of Rapa favours the differentiation of monocytes into immature DCs with a set of markers distinctive for myeloid DCs (CD11c and CD1c), but with a lack of monocyte marker CD14. Similarly, increased expression of CD1c was reported on Rapa-DC; it is possible that this could be correlated with regulatory cell infiltration (Monti et al. 2003).

The presence of Rapa during differentiation did not significantly affect the DC expression of surface markers related to T cell activation, such as MHC II, CD86 and CD40, except CD80. These findings are not in line with those of previous studies indicating that Rapa decreases the level of MHC II, CD86 and CD40 costimulatory molecules, with CD80 expression demonstrating considerable variance (Fedoric and Krishnan 2008; Haidinger et al. 2010; Monti et al. 2003). In addition, no changes in the expression of investigated costimulatory molecules during Rapa treatment were noted by Woltman et al. (2001). However, this variation may due to the different cell culture conditions, i.e. where Rapa was added after DC differentiation.

Our present findings indicate that the Rapa-DC culture displayed a significant decrease of CD80 expression. CD80 and CD86 are ligands of CD28 and cytotoxic T-lymphocyte-associated protein 4 (CTLA-4) receptors on T cells (Hubo et al. 2013). CD80 has greater affinity for CTLA-4 (Sansom and Walker 2006): Signalling by the CD80/CTLA-4 pathway can promote the generation and suppressive activity of Tregs, and signalling by CD86/CD28 induces T cell activation (Bhatia et al. 2006; Hubo et al. 2013; Pletinckx et al. 2011; Sansom and Walker 2006; Zheng et al. 2004). Experimental transplantology models suggest that CTLA-4 signalling is needed for immune tolerance induction and can prevent graft rejection (Judge et al. 1999; Yamada et al. 2001), whereas DC with CD80 expression but without CD86 can decrease rejection to xeno-islets (Ke et al. 2016).

The presence of CsA during DC differentiation resulted in increased CD86 and CD40 expression as well as decreased expression of CD80. Both CD86 and CD40 are involved in enhancing T cell activation (Hubo et al. 2013). The obtained results may suggest that CsA do not maintain the tolerogenic phenotype of DCs. Although the effects of CsA on CD86 and CD40 expression were not confirmed by previous studies (Duperrier et al. 2002; Fedoric and Krishnan 2008; Szabo et al. 2001; Woltman et al. 2000), this may be due to the different concentration of CsA used between the studies.

DC differentiation in the presence of Rapa resulted in a decreased percentage of DCs with TLR2, TLR4 and CCR7 expression. Many of the studies indicate that increased TLR2 and TLR4 expression on mononuclear cells may contribute to allograft rejection (Castillo et al. 2010; Deng et al. 2007; McDaniel et al. 2010; Methe et al. 2004; Testro et al. 2011). Therefore, the decreased TLR2 and TLR4 expression observed on Rapa-treated DCs may protect the organ recipient from rejection, by minimizing the response of DCs to specified ligands and subsequent T cell activation. Moreover, Rapa-DC can be less sensitive to activation signals through TLR, as Rapa may be able to induce the generation of maturation-resistant DCs. However, TLR signalling increases DC migration toward lymph nodes for antigen presentation (Howell et al. 2014). The decreased expression of CCR7 on Rapa-DC may result in reduced induction of immune response due to weak antigen presentation and DC migration. Those results are correlated with decreased expression of TLR2 and TLR4 as well as of the costimulatory molecules typical for immature DCs.

The elevated percentages of CD83-positive Rapa-DC and CsA-DC could act as a balance to the tolerogenic potential of Rapa and CsA, as CD83 is the marker for mature DCs and can act as an essential enhancer during T cell activation (Prechtel and Steinkasserer 2007). However, Kryczanowsky et al. (2016), report that tolDCs with high CD83 expression generated stronger regulatory function of induced Tregs than DCs with low CD83 expression. In other studies, the extracellular CD83 domain, as a soluble protein, inhibited DC maturation and T cell proliferation via DCs, thus demonstrating the strong immunosuppressive activity of CD83 (Fujimoto and Tedder 2006; Lechmann et al. 2001, 2002). It has been used in transplantation, where treatment with soluble CD83 has induced tolDCs that may promote tolerance and prevent allograft rejection (Ge et al. 2010; Lan et al. 2010; Xu et al. 2007; Yang et al. 2015).

The presence of Rapa or CsA during DC differentiation did not affect the production of anti-inflammatory cytokines (IL-4, TGF-β); however, the IL-10 level was lowered in the Rapa-treated culture. Comparable outcomes were previously observed in mouse DC cultures (Turnquist et al. 2010). Similarly, no significant differences were observed in pro-inflammatory cytokine production. In addition, the secretion of IL-12p70 was not detected in any cases. Our findings are in line with previous reports (Abdul et al. 2008; Ohtani et al. 2008; Sauma et al. 2003; Szabo et al. 2001; Turnquist et al. 2010). A common feature of tolerogenic DCs is the lack of IL-12. The obtained results could suggest that for DC tolerogenic properties to be maintained, cytokine levels may need to remain at those observed for immature DCs (Morelli and Thomson 2007; Steinman et al. 2003). In addition, some studies suggest that anti-inflammatory cytokine production is not needed to ensure the tolerogenic potential of Rapa-DC and CsA-DC (Monti et al. 2003; Naranjo-Gómez et al. 2011).

In our present study, both Rapa-DC and CsA-DC showed decreased phagocytic capacity and DEC205 expression. Comparable outcomes have also been observed in mouse and human DC studies (Abdul et al. 2008; Hackstein et al. 2002; Monti et al. 2003; Tajima et al. 2003). Decreased phagocytosis is a very important property of tolDCs, because it may also decrease the probability of T cell activation (Monti et al. 2003).

To confirm whether Rapa or CsA may generate maturation-resistant tolDCs, the study also evaluated phenotype and level of cytokine production by LPS-activated DCs differentiated in the presence of drugs. In addition, we also examined whether the presence of immunosuppressive agents during activation alone may generate maturation-resistant tolDCs. Our results indicate that the presence of Rapa or CsA during DC differentiation had a stronger influence on surface receptor expression and cytokine production than the presence of immunosuppressants during DC activation; this is in line with previous reports about Rapa (Fedoric and Krishnan 2008; Haidinger et al. 2010; Monti et al. 2003).

Our findings demonstrate that the presence of Rapa or CsA during DC differentiation, in particular Rapa, can induce generation of partly maturation-resistant DCs after LPS activation. LPS-activated Rapa-DC demonstrated decreased expression of MHC II and CD40, and decreased secretion of IL-10, TGF-β and IL-6. IL-12p70 was undetectable, which is in line with previous reports demonstrating the absence of IL-12, or decreased production, by activated Rapa-DC (Chen et al. 2004; Horibe et al. 2008; Monti et al. 2003; Naranjo-Gómez et al. 2011; Pino-Lagos et al. 2010; Sauma et al. 2003; Szabo et al. 2001;). Studies on animal models confirmed that the presence of Rapa during DC differentiation results in the generation of maturation-resistant DCs after LPS activation (Hackstein et al. 2003; Horibe et al. 2008; Taner et al. 2005). Furthermore, an infusion of Rapa-DC after composite tissue transplantation can prevent graft rejection (Ikeguchi et al. 2008). Similar findings were noted by Haidinger et al. (2010) in human DCs, where MHC II expression and IL-6 and IL-10 production were all decreased. In the case of CsA, while they do not appear to influence human DC activation with LPS (Szabo et al. 2001; Woltman et al. 2000), some mouse-based studies have found it to influence DC maturation (Pino-Lagos et al. 2010; Sauma et al. 2003).

An interesting observation is the increase in CCR7 expression associated with LPS-activated Rapa-DC. This is an important consideration in the clinical application of tolDCs, as CCR7-dependent migration toward secondary lymphoid organs is required for efficient induction of functional Tregs. Hence, the potential for migration of Rapa-DC may suggests that Rapa has immunosuppressive potential, as confirmed previously (Adnan et al. 2016; Förster 2008; Stallone et al. 2016). It can also be indicative of the generation of semi-mature DCs, cells which may induce Tregs (Lutz et al. 2002; Lutz 2012). Therefore, increasing the CCR7 expression on DCs can play an important role in tolerance promotion. Comparable outcomes were observed in various DC culture models. Rapa treatment was found to enhance the migration of activated DCs, or at least not inhibit it; this migration was accompanied by CCR7 expression comparable to control values or higher (Adnan et al. 2016; Boks et al. 2012; Sordi et al. 2006; Stallone et al. 2016; Taner et al. 2005; Turnquist et al. 2007). In a study of LPS-activated DCs, Sordi et al. (2006) attribute CCR7 up-regulation to inhibition of IL-10 production, and suggest that IL-10 may inhibit CCR7 expression. Assuming this is the case, the increase in CCR7 expression observed in LPS-activated Rapa-DC in the present study may be due, at least partially, to a fall in IL-10 production.

The presence of Rapa during DC differentiation did not influence their potential for T cell activation, No changes in the expression of activation markers or percentage of proliferating T cells were observed. Previous studies indicate that immature Rapa-DC, both human and mouse, have a limited potential to induce T cell proliferation (Monti et al. 2003; Taner et al. 2005; Turnquist et al. 2007, 2010).

In addition, a significant decrease of PD-L1 expression was observed on immature Rapa-DC, which may suggest that Rapa does not influence the allostimulatory capacity of DCs. Previous studies confirm that PD-L1 expression is decreased on the surface of DCs during Rapa treatment (Boks et al. 2012; Haidinger et al. 2010; Macedo et al. 2013). However, our present findings indicate that Rapa decreased IL-6 and IL-10 production by immature and LPS-activated DC. This may be associated with the low level of PD-L1 expression on Rapa-DC, because increased PD-L1 expression is correlated with high IL-6 and IL-10 production (Sumpter and Thomson 2011; Wolfle et al. 2011). Rosborough et al. (2013) report that Rapa inhibited mammalian target of rapamycin complex 1 and caused atypical reduction of IL-10 synthesis and PD-L1 expression on mouse DCs. It is possible that tolerogenic Rapa activity is not correlated with changes of PD-L1 expression.

In contrast to Rapa, CsA was found to inhibit T cell activation potential by the studied DC, which is due to the lower percentage of proliferating T cells and CD3^+^CD69^+^ T cells in the CsA-DC culture. These results are in line with those of previous studies (Duperrier et al. 2002; Geng et al. 2008; Matsue et al. 2002; Szabo et al. 2001). Similarly, PD-L1 expression was significantly reduced on immature DC differentiated in the presence of CsA. Unfortunately, no data are available concerning the effects of CsA on PD-L1 expression on DCs. Only Geng et al. (2008) indicate that CsA does not appear to have an influence on PD-L1 expression in mouse DCs. Based on those results, it is likely that the PD-1/PD-L1 pathway inhibition mechanism does not play a crucial role in the tolerogenic influence of CsA-DC on T cell activation.

In addition, our findings indicate that DCs differentiated in the presence of immunosuppressive agents reduced the susceptibility of T cells to apoptosis via decreased CD95 expression on the T cell surface; however, they did not affect cell viability or apoptosis induction among T cells. Stenger et al. (2014) report that murine Rapa-DC promotes apoptosis of alloreactive T cells via induction of IFN-γ production by the cells. Moreover, this induction also entails increased CD95 expression on T cells. The interaction between CD95 and CD95L is a significant mechanism of apoptosis promotion by DCs. Our results are in line with other studies indicating that Rapa-DC did not affect T cell apoptosis induction (Naranjo-Gómez et al. 2011; Taner et al. 2005). However, no previous studies have examined this aspect of CsA-DC.

Our data confirm that Rapa can influence the ability of DCs to induce Tregs, which is in line with previous outcomes (Naranjo-Gómez et al. 2011; Turnquist et al. 2007, 2010). In our present study, CsA-DC also showed the ability to generate Treg cells; however, lower CD25 and Foxp3 expression was observed compared to T cells cultured with Rapa-DC. These results are in contrast to those of Fedoric and Krishnan (2008), who demonstrated that CsA-DC are unable to induce Treg generation, and those of Pino-Lagos et al. (2010), who found that murine CsA-treated DCs reduce the proliferation of Tregs. Any differences between studies can be attributed to heterogeneity regarding experimental protocols and drug concentrations.

Interestingly, the combination of higher CD86 and lower CD80 expression on CsA-DC surface did not influence the Treg generation potential of the DCs. Similarly, Pletinckx et al. (2011), suggest that DCs with moderate, or even high expression of CD80/CD86 costimulatory molecules are effective Treg inductors. This may suggest that the level of CD80/CD86 expression may not be crucial for Treg generation by DCs. In contrast, our present findings indicate that the Rapa-DCs demonstrating a similar CD80/CD86 expression profile to control immature DCs, induced a higher percentage of Tregs than the CsA-DCs.

Our results indicate that DCs differentiated in the environment of Rapa or CsA did not increase the percentage of studied CD8^+^ T cells, while coculture with CsA-DC significantly decreased the percentage of CD8^+^CD25^+^CD28^+^ T cells. This could suggest that neither studied immunosuppressant affects the ability of DCs to induce generation of CD8^+^ T cells with regulatory function.

To assess Th2 cell activation and the allostimulatory capacity of DCs, it was also important to evaluate production of cytokines by T cells during DC-T cell interactions. While previous studies report decreased production of IL-2 and IFN-γ, characteristic of Th1, and IL-4, IL-5, IL-10 characteristic of Th2, most of them were observed in studies of activated DC (Monti et al. 2003; Naranjo-Gómez et al. 2011; Taner et al. 2005; Turnquist et al. 2010; Wang et al. 2009). A review of the few existing investigations of immature Rapa-DC cells suggests that they induce decreased production of IL-10 by T cells (Taner et al. 2005) with no effect on IFN-γ and IL-4 production (Turnquist et al. 2010). Only Stenger et al. (2014), report a significant elevation of IFN-γ synthesis by T cells, but this was associated with the interaction with IL-12^hi^ Rapa-DC.

In line with Matsue et al. (2002) and Tajima et al. (2003), our results also indicate decreased production of IL-2 and IFN-γ by T cells stimulated with CsA-DC, as well as lower production of the Th2-characteristic cytokines IL-5 and IL-13. This confirms the observations of Abdul et al. (2008), that neither CsA nor Rapa influence the ability of DCs to induce Th2 cells activation, and that they can even inhibit it. Moreover, the lack of IL-12 production by Rapa-DC and CsA-DC, a cytokine crucial in the development of the Th1 response, resulted in a weakening of the ability of Rapa-DC and CsA-DC to polarize T cells into a Th1 subpopulation; this may indicate a decreased allostimulatory ability by these DCs, and can be one of mechanisms underlying their tolerogenicity.

In conclusion, our results demonstrate that Rapa, in contrast to CsA, is effective at generating maturation-resistant DCs with tolerogenic properties, such as low expression of molecules associated with antigen presentation and T cell activation, low expression of TLR2 and TLR4 and decreased phagocytic capacity. Such Rapa-DCs induce Treg generation. In the case of CsA, it is not efficient at generating maturation-resistant tolDCs, but affects the ability of these cells to decrease Th1-characteristic cytokine production and induce Treg generation. Moreover, the presence of Rapa or CsA only during DC activation with LPS did not affect the generation of maturation-resistant DCs.

## Supplementary Information

Below is the link to the electronic supplementary material.

Supplementary file1 (TIF 19499 KB)

Supplementary file2 (TIF 556 KB)

Supplementary file3 (TIF 259 KB)

Supplementary file4 (TIF 257 KB)

Supplementary file5 (DOCX 23 KB)

## Data Availability

Not applicable.

## Abbreviations

APC: Allophycocyanin
CCR: C–C chemokine receptor
CFSE: Carboxyfluorescein succinimidyl ester
CsA: Cyclosporine A
CTLA-4: Cytotoxic T-lymphocyte-associated protein 4
IFN: Interferon
IL: Interleukin
DCs: Dendritic cells
ELISA: Enzyme-linked immunosorbent assay
FITC: Fluorescein isothiocyanate
FMO: Fluorescence minus one
GM-CSF: Granulocyte–macrophage colony-stimulating factor
LPS: Lipopolysaccharide
MFI: Mean fluorescence intensity
MHC: Major histocompatibility complex
MLR: Mixed lymphocyte reaction
PBMC: Peripheral blood mononuclear cells
PE: Phycoerythrin
Rapa: Rapamycin
TGF: Transforming growth factor
TLR: Toll-like receptor
Tregs: T regulatory cells
