# Supplementary figures and images for: Cyclosporine A, in Contrast to Rapamycin, Affects the Ability of Dendritic Cells to Induce Immune Tolerance Mechanisms
Source: Arch Immunol Ther Exp (Warsz). 2021 Oct 10;69(1):27. doi: 10.1007/s00005-021-00632-7 (PMC8502748; doi:10.1007/s00005-021-00632-7)

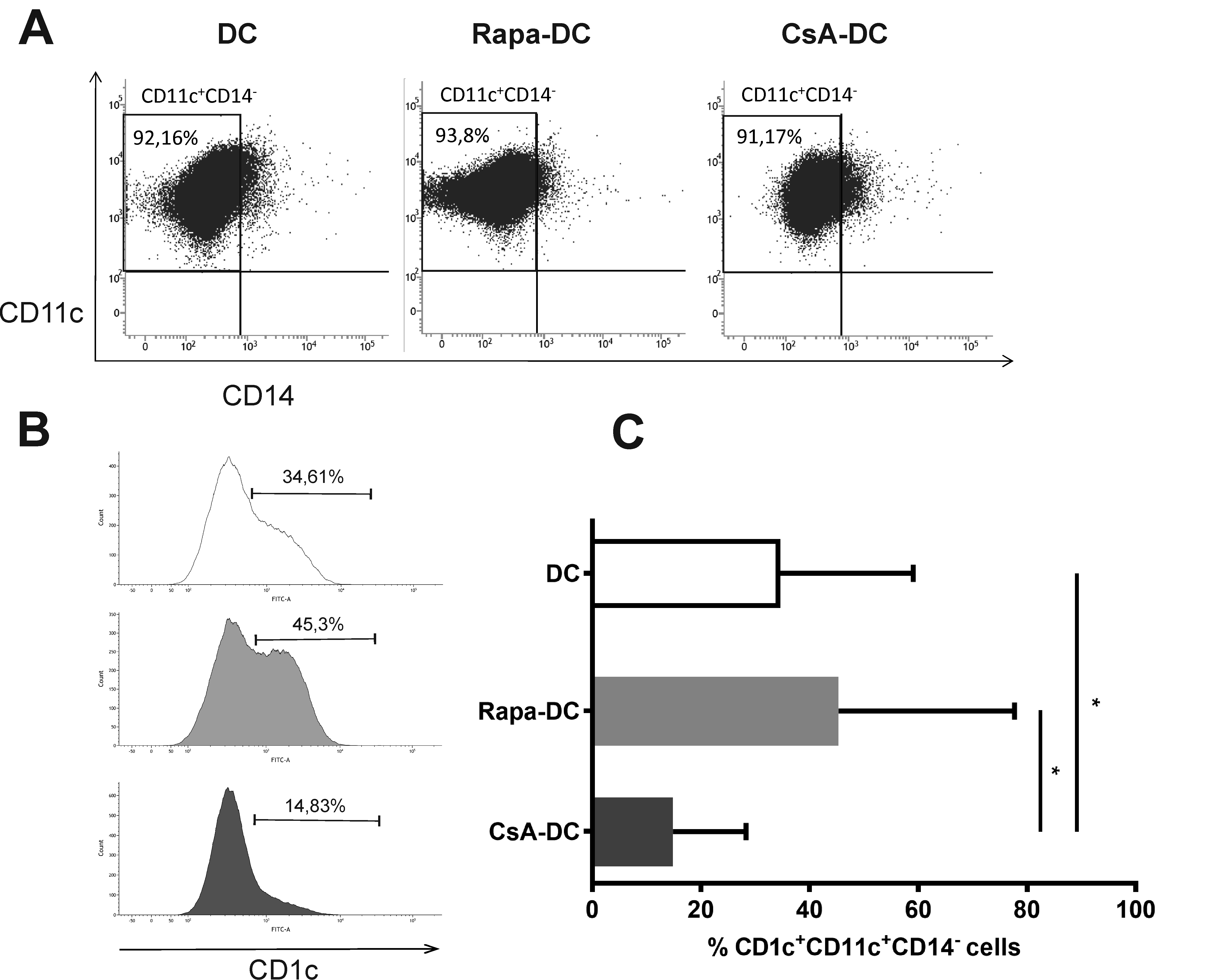

Supplement: Supplementary file 1 — Supplementary file1 (TIF 19499 KB) [file 5_2021_632_MOESM1_ESM.tif]

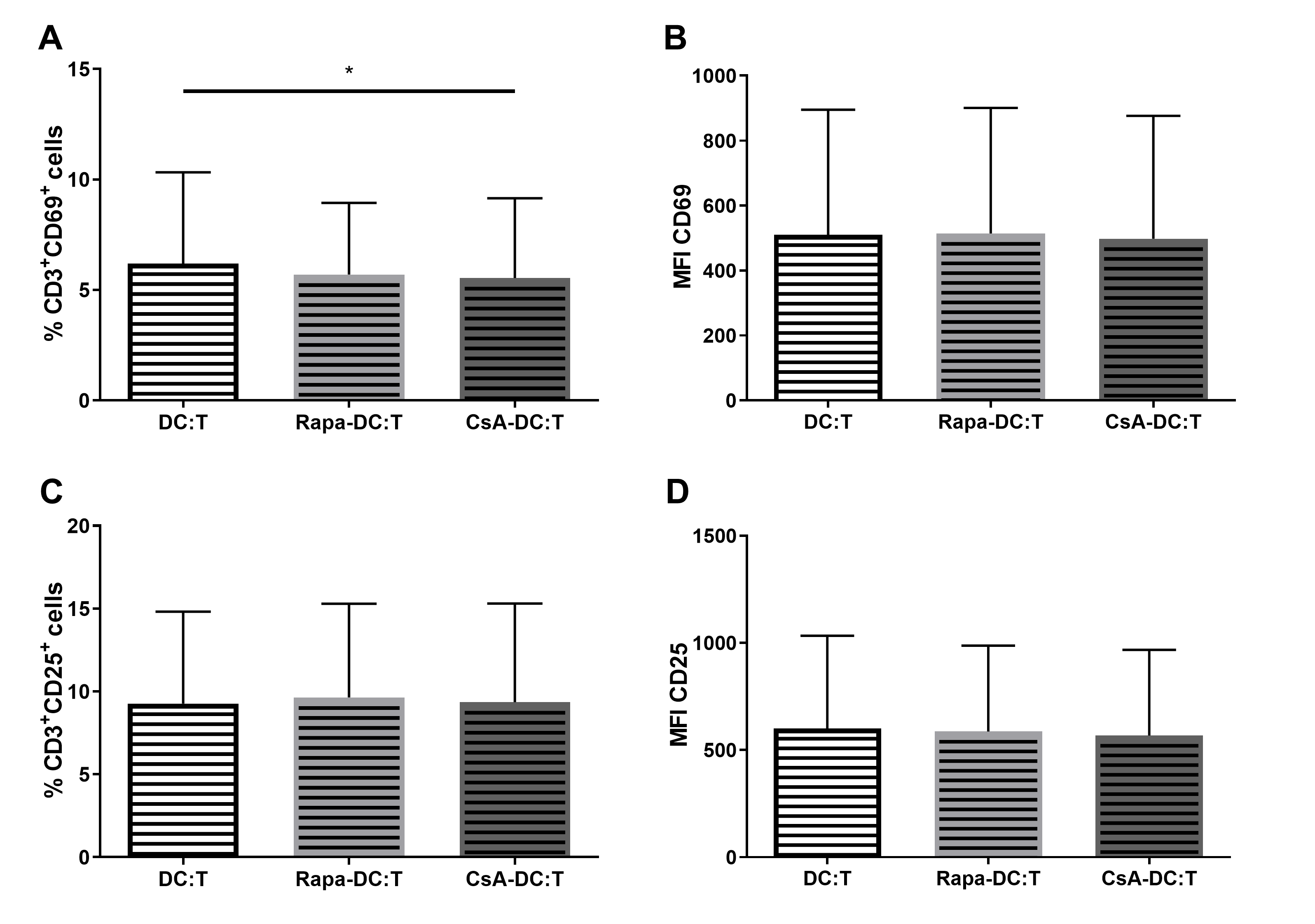

Supplement: Supplementary file 2 — Supplementary file2 (TIF 556 KB) [file 5_2021_632_MOESM2_ESM.tif]

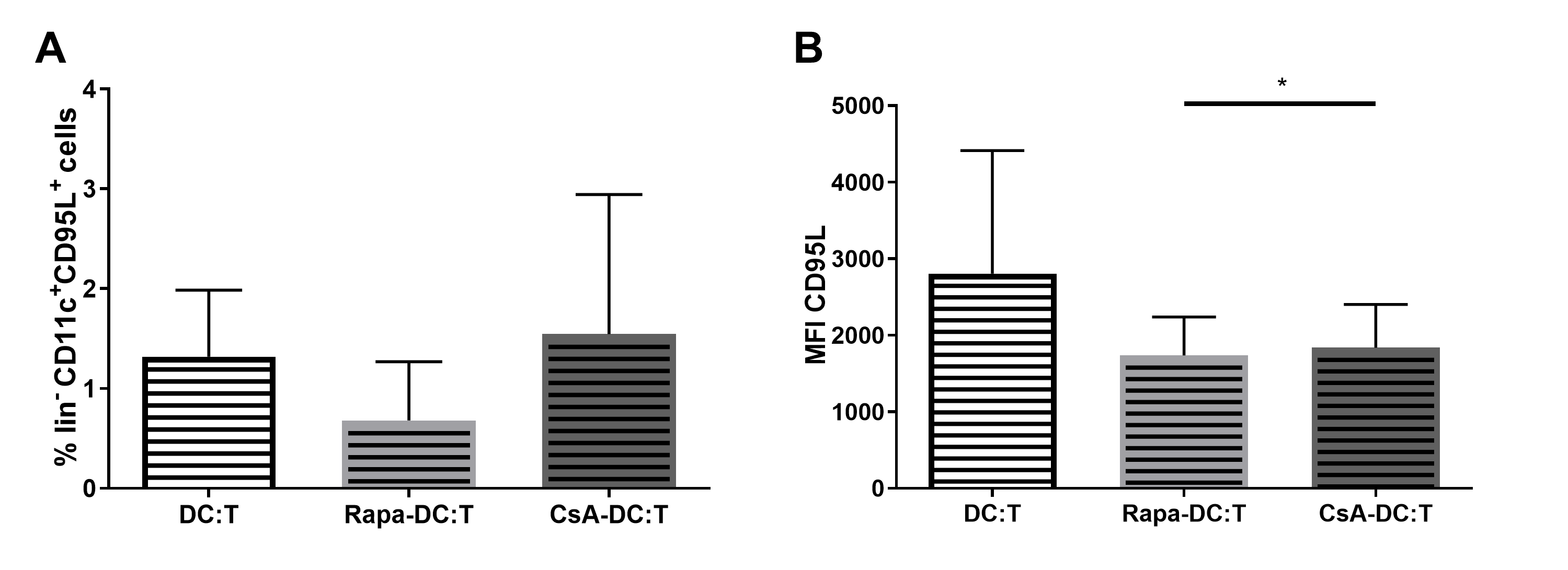

Supplement: Supplementary file 3 — Supplementary file3 (TIF 259 KB) [file 5_2021_632_MOESM3_ESM.tif]

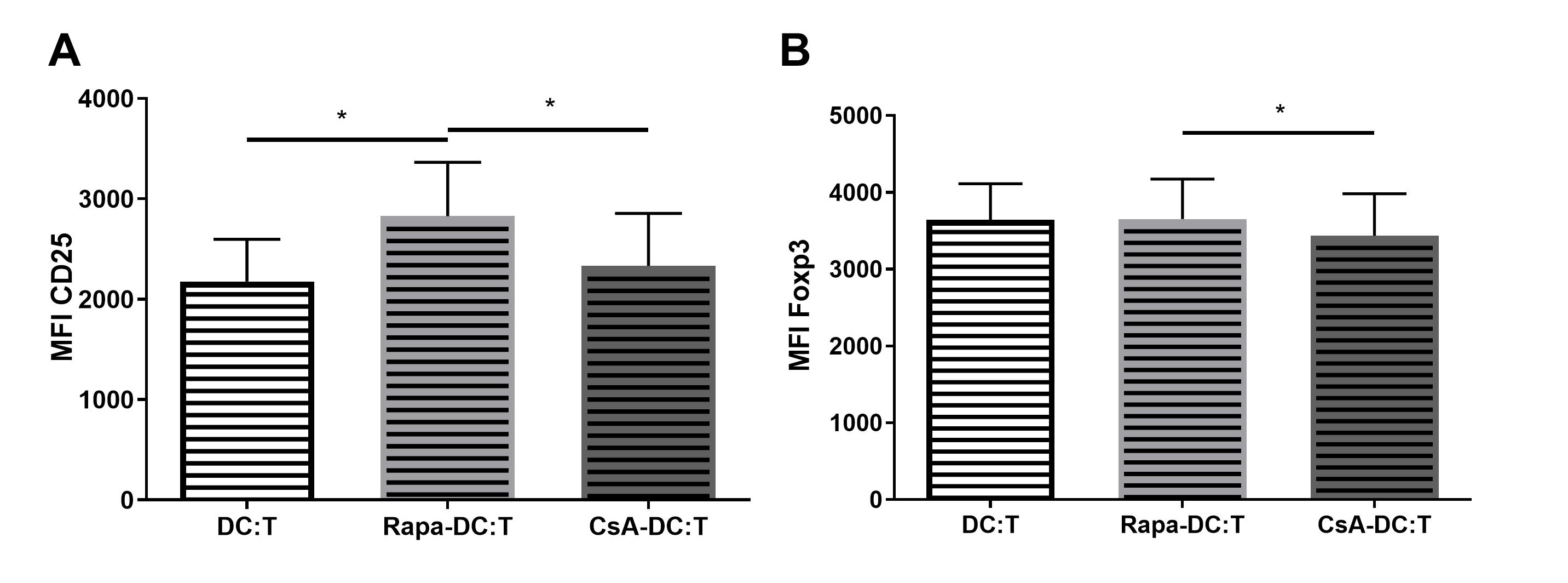

Supplement: Supplementary file 4 — Supplementary file4 (TIF 257 KB) [file 5_2021_632_MOESM4_ESM.tif]
