# Supplementary material for: Cyclosporine A, in Contrast to Rapamycin, Affects the Ability of Dendritic Cells to Induce Immune Tolerance Mechanisms
Source: Arch Immunol Ther Exp (Warsz). 2021 Oct 10;69(1):27. doi: 10.1007/s00005-021-00632-7 (PMC8502748; doi:10.1007/s00005-021-00632-7)
Supplement: Supplementary file 5 — Supplementary file5 (DOCX 23 KB) [file 5_2021_632_MOESM5_ESM.docx]

**Fig. S1** Effects of Rapa and CsA on DC differentiation. Immature DCs were differentiated in the environment of immunosuppressive agents: Rapa (Rapa-DC) or CsA (CsA-DC) and without drugs (DC). Percentage of CD11c^+^CD14^–^ DCs and followed CD1c expression was determined by flow cytometry. The representative plots show the average percentage of CD11c^+^CD14^–^ cells (A) and histograms present gating for CD11c^+^CD14^–^CD1c^+^ cells (B). The graphs display the averages ± SD of the percentages of CD11c^+^CD14^–^CD1c^+^ cells from nine different donors (C). **p*≤0.05; *p* values were calculated by Wilcoxon matched pair test.

**Fig. S2** Effects of Rapa-DC and CsA-DC on the expression of CD25 and CD69 on T cells. Immature DCs, Rapa-DC and CsA-DC were cocultured with T cells. Expression of CD25 and CD69 was determined by flow cytometry. Results are the averages ± SD of the percentage of positively labelled CD3^+^CD69^+^ (A) and CD3^+^CD25^+^ (C) T cells and mean fluorescence intensity (MFI; B, D) from 13 different donors. **p*≤0.05; *p* values were calculated by Wilcoxon matched pair test.

**Fig. S3** CD95L expression on DCs in MLR cultures. Immature DCs, Rapa-DC and CsA-DC were cocultured with T cells. Expression of CD95L was determined by flow cytometry. Results are the averages ± SD of the percentage of lin^–^CD11c^+^CD95L^+^ DCs (A) and CD95L mean fluorescence intensity (MFI; B) from eight different donors. **p*≤0.05; *p* values were calculated by Wilcoxon matched pair test.

**Fig. S4** Effects of Rapa-DC and CsA-DC on the expression of CD25 and Foxp3 on CD4 Tregs. Immature DCs, Rapa-DC and CsA-DC were cocultured with T cells. Expression of CD25 and Foxp3 on CD4^+^CD25^high^Foxp3^+^ Tregs was determined by flow cytometry. Results are the averages ± SD of the mean fluorescence intensity (MFI) of CD25 (A) and Foxp3 (B) from ten different donors. **p*≤0.05; *p* values were calculated by Wilcoxon matched pair test.
